# Supplementary material for: Resolving the full spectrum of human genome variation using Linked-Reads
Source: Genome Res. 2019 Apr;29(4):635–45. doi: 10.1101/gr.234443.118 (PMC6442396; doi:10.1101/gr.234443.118)
Supplement: Supplemental Material [file supp_gr.234443.118_Supplemental_longranger-2.2.zip › longranger-2.2.tar.bz2!/lr2.2-public/lib/stan/cmdstan/stan/doc/api/html/math_2matrix_2log__determinant__ldlt_8hpp_source.html]

Stan: src/stan/math/matrix/log\_determinant\_ldlt.hpp Source File


|  |  |
| --- | --- |
|  | Stan  2.5.0  probability, sampling & optimization |


- Main Page
- Related Pages
- Namespaces
- Classes
- Files

- File List
- File Members

All Classes Namespaces Files Functions Variables Typedefs Enumerations Enumerator Friends Macros Pages

- src
- stan
- math
- matrix

log\_determinant\_ldlt.hpp

Go to the documentation of this file.

1 #ifndef STAN\_\_MATH\_\_MATRIX\_\_LOG\_DETERMINANT\_LDLT\_HPP

2 #define STAN\_\_MATH\_\_MATRIX\_\_LOG\_DETERMINANT\_LDLT\_HPP

3

4 #include <stan/math/matrix/LDLT\_factor.hpp>

5

6 namespace stan {

7  namespace math {

8

9  // Returns log(abs(det(A))) given a LDLT\_factor of A

10  template<int R, int C, typename T>

11  inline T

12  log\_determinant\_ldlt(stan::math::LDLT\_factor<T,R,C> &A) {

13  return A.log\_abs\_det();

14  }

15

16  }

17 }

18 #endif

stan::math::LDLT\_factor< T, R, C >::log\_abs\_det

T log\_abs\_det() const

**Definition:** LDLT\_factor.hpp:81

stan::math::LDLT\_factor< T, R, C >

LDLT\_factor is a thin wrapper on Eigen::LDLT to allow for reusing factorizations and efficient autodi...

**Definition:** LDLT\_factor.hpp:56

stan::math::log\_determinant\_ldlt

T log\_determinant\_ldlt(stan::math::LDLT\_factor< T, R, C > &A)

**Definition:** log\_determinant\_ldlt.hpp:12

LDLT\_factor.hpp


---

[ Stan Home Page ]

*© 2011–2014,
Stan Development Team.*
